# Public Interest in Immunity and the Justification for Intervention in the Early Stages of the COVID-19 Pandemic: Analysis of Google Trends Data

**DOI:** 10.2196/26368

**Published:** 2021-06-18

**Authors:** Jinhee Lee, Yunna Kwan, Jun Young Lee, Jae Il Shin, Keum Hwa Lee, Sung Hwi Hong, Young Joo Han, Andreas Kronbichler, Lee Smith, Ai Koyanagi, Louis Jacob, SungWon Choi, Ramy Abou Ghayda, Myung-Bae Park

**Affiliations:** 1 Department of Psychiatry Yonsei University Wonju College of Medicine Wonju Republic of Korea; 2 Department of Psychology Duksung Women’s University Seoul Republic of Korea; 3 Department of Nephrology Yonsei University Wonju College of Medicine Wonju Republic of Korea; 4 Department of Pediatrics Yonsei University College of Medicine Seoul Republic of Korea; 5 Department of Pediatrics Samsung Changwon Hospital Sungkyunkwan University School of Medicine Changwon Republic of Korea; 6 Department of Internal Medicine IV, Nephrology and Hypertension Medical University Innsbruck Innsbruck Austria; 7 The Cambridge Centre for Sport and Exercise Sciences Anglia Ruskin University Cambridge United Kingdom; 8 Parc Sanitari Sant Joan de Déu/Centro de Investigación Biomédica en Red de Salud Mental Universitat de Barcelona Barcelona Spain; 9 Catalan Institution for Research and Advanced Studies Barcelona Spain; 10 Faculty of Medicine University of Versailles Saint-Quentin-en-Yvelines Paris France; 11 Department of Global Health and Population Harvard T.H. Chan School of Public Health Boston, MA United States; 12 Urology Institute University Hospitals Case Western Reserve University Cleveland, OH United States; 13 Department of Gerontology Health and Welfare Pai Chai University Daejeon Republic of Korea

**Keywords:** COVID-19, social big data, infodemiology, infoveillance, social listening, immune, vitamin, big data, public interest, intervention, immune system, immunity, trends, Google Trends, internet, digital health, web-based health information, correlation, social media, infectious disease

## Abstract

**Background:**

The use of social big data is an important emerging concern in public health. Internet search volumes are useful data that can sensitively detect trends of the public's attention during a pandemic outbreak situation.

**Objective:**

Our study aimed to analyze the public’s interest in COVID-19 proliferation, identify the correlation between the proliferation of COVID-19 and interest in immunity and products that have been reported to confer an enhancement of immunity, and suggest measures for interventions that should be implemented from a health and medical point of view.

**Methods:**

To assess the level of public interest in infectious diseases during the initial days of the COVID-19 outbreak, we extracted Google search data from January 20, 2020, onward and compared them to data from March 15, 2020, which was approximately 2 months after the COVID-19 outbreak began. In order to determine whether the public became interested in the immune system, we selected *coronavirus*, *immune*, and *vitamin* as our final search terms.

**Results:**

The increase in the cumulative number of confirmed COVID-19 cases that occurred after January 20, 2020, had a strong positive correlation with the search volumes for the terms *coronavirus* (*R*=0.786; *P*<.001), *immune* (*R*=0.745; *P*<.001), and *vitamin* (*R*=0.778; *P*<.001), and the correlations between variables were all mutually statistically significant. Moreover, these correlations were confirmed on a country basis when we restricted our analyses to the United States, the United Kingdom, Italy, and Korea. Our findings revealed that increases in search volumes for the terms *coronavirus* and *immune* preceded the actual occurrences of confirmed cases.

**Conclusions:**

Our study shows that during the initial phase of the COVID-19 crisis, the public’s desire and actions of strengthening their own immune systems were enhanced. Further, in the early stage of a pandemic, social media platforms have a high potential for informing the public about potentially helpful measures to prevent the spread of an infectious disease and provide relevant information about immunity, thereby increasing the public’s knowledge.

## Introduction

Following the onset of an infectious pneumonia that could be traced to Wuhan, China, in 2019, the World Health Organization (WHO) announced that the cause of the pneumonia was a new type of coronavirus. The International Committee on Taxonomy of Viruses named it SARS-CoV-2 on on January 11, 2020, and the infectious pneumonia was referred to as COVID-19. As of December 2020, the virus has spread globally; the cumulative number of people infected with SARS-CoV-2 has reached approximately 67 million, and approximately 1,530,000 have died from the virus [1].

Since the year 2000, there have been attempts to use big data to better understand and solve challenges related to public health. Indeed, in the context of health care, the use of big data is an important emerging trend that is only likely to increase in importance over time. The Pillbox project of the National Laboratory of Medicine is one of the most representative examples of health care that uses big data to predict real-world situations. The Pillbox project is a service that provides information such as how to take various pills that are not well known by the public and their potential side effects. The use of such information not only improves consumer convenience but also saves health care expenditures with regard to economics through the gathering of information to estimate statistics, such as the causes of disease outbreaks, rates of spread, and distribution and regional growth [2,3]. Social big data refers to large volumes of data that relate to people or describe people’s behaviors and technology-mediated social interactions in the digital realm [4]. Internet search volume is a valid and effective tool for detecting public attention [5]. One of the most representative examples of using social big data for disease prediction is Google’s flu forecast. In 2009, Google predicted the spread of the flu 7 to 10 days earlier than the Centers for Disease Control and Prevention (CDC) based on users’ search records for the flu [6]. This prediction was confirmed in countries around the world, including South Korea, India, and China [7-9]. Predictive analyses that use social big data, which are not easily accessible in reality, help with making relatively accurate predictions of real-world events. In recent times, attempts have been made to further increase the precision of disease predictions [10,11]. Furthermore, predictive analyses were also used for the real-time monitoring of SARS (severe acute respiratory syndrome) and its transmissibility and for the identification of the natural history of emerging pathogens, such as the Ebola virus [12,13].

The spread of infectious diseases has led to increased interest in the human immune system among the public. During a pandemic, the public often becomes interested in how to strengthen their immune systems. Furthermore, the act of purchasing products that may strengthen one’s immune system is popular, even without clear evidence that shows whether these supplements are effective. However, there is almost no scientific proof for confirming this trend, and there is a need to confirm that such interest results in people actually searching more areas of the internet for products related to strengthening their immune system. Eventually, people are likely to respond to this lack of evidence by relying on existing common sense when it comes to maintaining health and hygiene. In this regard, a health literacy approach is needed during a crisis such as the COVID-19 pandemic. At the beginning of a pandemic, the public’s response to an infectious disease is the most sensitive, and people have a burgeoning interest in infectious diseases during this period. After the passage of a certain period of time, exhaustion and the adjustment to the social situation results in a relative decline in this interest [14].

Our study aims to analyze the public’s interest in the global pandemic and correlate internet search volumes for COVID-19–related terms with the public’s interest in the immune system and vitamins.

## Methods

### Data Extraction

To assess the public’s interest in infectious diseases during the initial days of the COVID-19 outbreak, we extracted Google Trends search data from January 20, 2020, onward and compared them to data from March 15, 2020, which was approximately 2 months after the COVID-19 outbreak began. Apart from in a few countries such as China and Japan, the Google search engine has an overwhelming dominance of market shares [15]. Google provides data on search word volumes through Google Trends, which provides usable data for specific countries and time periods. In this study, our unit of data was extracted from data on total worldwide numbers. In addition, to account for trends by country, we selected the United States and the United Kingdom for analysis, as these countries are the most representative countries of the English-speaking world that use the Google search engine. Moreover, we also studied Italy, which was the first European country to experience an explosion in the number of COVID-19 cases. The market share of Google in Italy in January 2020 was 98.9% [16]. Finally, we studied South Korea, which had the second highest COVID-19 spread rate in Asia and the fourth highest spread rate in the world at the time the Google Trends search was performed. On the date that the data were gathered, South Korea was ranked fourth in the world for COVID-19 case numbers, with 8000 cases. In South Korea, the domestic portal site Naver (Naver Corporation) has a much higher market share than Google. Therefore, additional data supplied by Naver Data Lab were used for cross-verification.

### Primary Keywords

We used the terms *covid*, *corona*, and *coronavirus* as search terms. In order to determine which of these words were the most commonly used, the search volume of each word was ascertained. According to our preliminary analysis of the data provided by Google Trends, the term *coronavirus* was searched twice more often than *corona* and 5 times more often than *covid*. Therefore, we selected *coronavirus* as our final search term.

### Searches Regarding Immunity and Products That Might Strengthen Immunity

In order to determine whether the public became interested in the immune system, we selected *immune* as a keyword. The term *immune* was strongly related to the term *immunity* and had an almost 2-fold higher search volume. Therefore, for the United States, United Kingdom, and Italy, the search term *immune* was studied, and in the case of South Korea, the term *myeonyeok*, which means “immunity” in Korean, was studied.

Following the outbreak of COVID-19, the public’s interest in products that can enhance one’s immune system increased dramatically. The Guardian in the United Kingdom and the Washington Post in the United States have both reported on the rising demand for vitamins and other products [17]. Vitamins are the most familiar to the public, are usually available over the counter, and are the most representative product that enhances the immune system. Therefore, in order to analyze the public’s actual interest in immune system strength, we selected the term *vitamin* as a keyword. For the United States and the United Kingdom, the term *vitamin* was used. For Italy, the term *vitamina* was used. Finally, for South Korea, the Korean-language spelling of the term *vitamin* was used.

### Data and Statistical Analysis

Search volumes were calculated for each period (1 week), and the search volume of the period with the highest search volume was 100, which normalized every other search volume value from 0 to 100. The unit of extraction was 1 week. In order to account for the spread of COVID-19, we used the cumulative number of confirmed cases. The official announcements made by the WHO and announcements from each country’s respective government were the most accurate indices for tracking the spread of SARS-CoV-2. The number of cumulative, confirmed cases was verified with data downloaded from Our World in Data [18] and additional data from COVID-19 situation reports issued by the WHO [1]. We conducted an analysis of the correlation between the number cumulative, confirmed COVID-19 cases and search terms. Up until the middle of January 2020, before COVID-19 had begun to spread on a large scale, the figure for confirmed cases and search volume was close to 0. In other words, the actual figure was 0 or 1; therefore, a correlation analysis of the entire year could lead to errors. As such, we selected countries that experienced some of the earliest COVID-19 outbreaks as our target countries. Moreover, we analyzed approximately 2 months’ worth of data, beginning from when COVID-19 cases first began to emerge globally during the week of January 20, 2020, up until March 15, 2020.

### Data Availability

Data can be downloaded from the Google Trends website. If the processed data are needed, the authors can be contacted to request the data.

## Results

**Figure 1:**
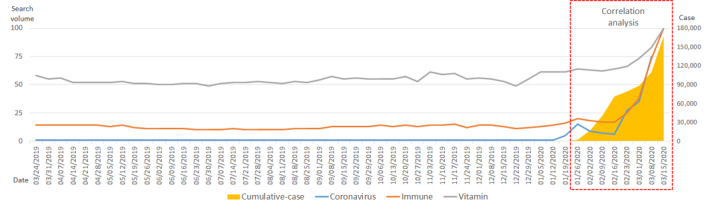
Worldwide trends in search volumes for the terms *coronavirus*, *immune*, and *vitamin*, and the number of cumulative, confirmed COVID-19 cases over the past year (from March 18, 2019, to March 15, 2020).

Up until the middle of January 2020, the number of confirmed COVID-19 cases remained extremely low worldwide. Afterward, beginning in the week of January 26, the number of confirmed cases began to increase rapidly, especially in China. The search volume for the term *coronavirus* began to increase earlier—beginning in the week of January 12—and the search volume increased rapidly during the week of February 16. For the past year, the search volume for the term *immune* has consistently been between 10 and 20. After January 12, the search volume increased steadily, and from February 16 onward, the search volume increased rapidly. Throughout 2020, the search volume for the term *vitamin* was at a level that slightly exceeded 50. However, this began to increase after February. The increase in the number of cumulative, confirmed cases of COVID-19 after January 20 had a strong positive correlation with the search volumes for the terms *coronavirus* (*R*=0.786; *P*<.001), *immune* (*R*=0.745; *P*<.001), and *vitamin* (*R*=0.778; *P*<.001), and the correlations between variables were all mutually statistically significant (Figure 1, Table 1).

**Table 1:** Correlations among search volumes for the terms *coronavirus*, *immune*, and *vitamin*, and the actual number of cumulative, confirmed COVID-19 cases worldwide (over 56 days).

Variable			Cumulative case numbers		Coronavirus		Immune		Vitamin		
**Cumulative case numbers**											
	*R*	1		0.786		0.745		0.778			
	*P* value	—^a^		<.001		<.001		<.001			
**Coronavirus**											
	*R*	0.786		1		0.979		0.932			
	*P* value	<.001		—		<.001		<.001			
**Immune**											
	*R*	0.745		0.979		1		0.943			
	*P* value	<.001		<.001		—		<.001			
**Vitamin**											
	*R*	0.778		0.932		0.943		1			
	*P* value	<.001		<.001		<.001		—			

^a^Not applicable.

**Figure 2:**
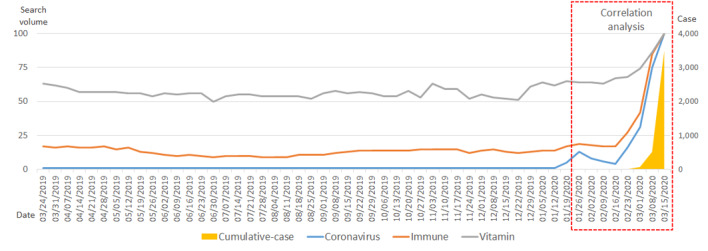
Trends in search volumes for the terms *coronavirus*, *immune*, and *vitamin*, and the number of cumulative, confirmed COVID-19 cases in the United States over the past year (March 18, 2019, to March 15, 2020).

With respect to the United States, up until February 2020, the number of cumulative, confirmed cases remained low (10 people). This number began increasing in the latter half of February and began increasing rapidly in March. The search volume for the term *coronavirus* began increasing earlier—beginning in the week of January 12—and it began to increase rapidly in the week of February 16. The search volume for the term *immune* began to rapidly increase on February 16. Over the past year, the search volume for the term *vitamin* was at a level that slightly exceeded 50. This number increased slightly after December 2019 and increased more rapidly in March 2020. The increase in the number of cumulative, confirmed cases after January 20 had a strong positive correlation with the search volumes for the terms *coronavirus* (*R*=0.921; *P*<.001), *immune* (*R*=0.890; *P*<.001), and *vitamin* (*R*=0.913; *P*<.001), and the correlations between variables were all mutually statistically significant (Figure 2, Table 2).

**Table 2:** Correlations among search volumes for the terms *coronavirus*, *immune*, and *vitamin*, and the actual number of cumulative, confirmed COVID- 19 cases in the United States (over 56 days).

Variable		Cumulative case numbers	Coronavirus	Immune	Vitamin
**Cumulative case numbers**					
	*R*	1	0.921	0.890	0.913
	*P* value	—^a^	<.001	<.001	<.001
**Coronavirus**					
	*R*	0.921	1	0.983	0.952
	*P* value	<.001	—	<.001	<.001
**Immune**					
	*R*	0.890	0.983	1	0.946
	*P* value	<.001	<.001	—	<.001
**Vitamin**					
	*R*	0.913	0.952	0.946	1
	*P* value	<.001	<.001	<.001	—

^a^Not applicable.

**Figure 3:**
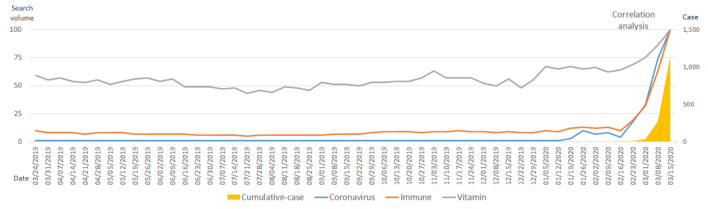
Trends in search volumes for the terms *coronavirus*, *immune*, and *vitamin*, and the number of cumulative, confirmed COVID-19 cases in the United Kingdom over the past year (March 18, 2019, to March 15, 2020).

In the United Kingdom, the number of confirmed cases increased rapidly after March 2020. Increases in search volumes for the terms *coronavirus*, *immune*, and *vitamin* followed a trend that was similar to those observed for the United States and the rest of the world. The number of cumulative, confirmed cases had a strong positive correlation with the search volumes for the terms *coronavirus* (*R*=0.931; *P*<.001), *immune* (*R*=0.962; *P*<.001), and *vitamin* (*R*=0.801; *P*<.001), and the correlations between variables were mutually statistically significant (Figure 3, Table 3).

**Table 3:** Correlations among search volumes for the terms *coronavirus*, *immune*, and *vitamin*, and the actual number of cumulative, confirmed COVID-19 cases in the United Kingdom (over 56 days).

Variable		Cumulative case numbers		Coronavirus	Immune		Vitamin
**Cumulative case numbers**							
	*R*	1	0.931		0.962	0.801	
	*P* value	—^a^	<.001		<.001	<.001	
**Coronavirus**							
	*R*	0.931	1		0.984	0.842	
	*P* value	<.001	—		<.001	<.001	
**Immune**							
	*R*	0.962	0.984		1	0.847	
	*P* value	<.001	<.001		—	<.001	
**Vitamin**							
	*R*	0.801	0.842		0.847	1	
	*P* value	<.001	<.001		<.001	—	

^a^Not applicable.

**Figure 4:**
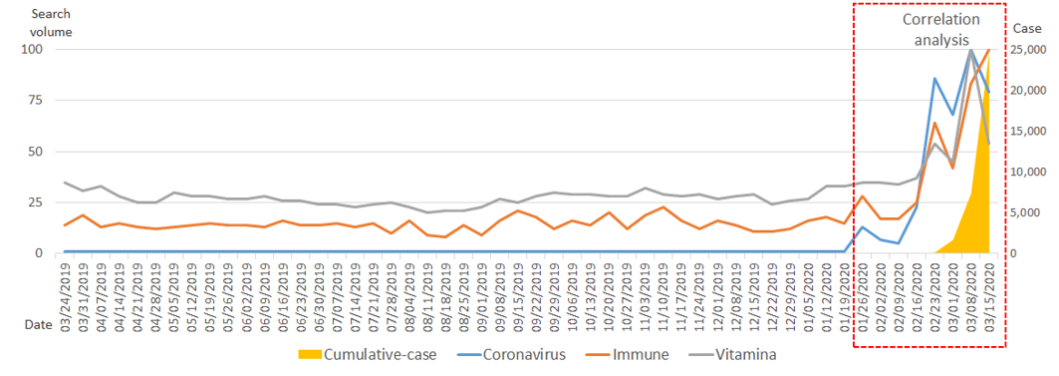
Trends in search volumes for the terms *coronavirus*, *immune*, and *vitamin*, and the number of cumulative, confirmed COVID-19 cases in Italy over the past year (March 18, 2019, to March 15, 2020).

In Italy, the number of confirmed cases started to rise in the second half of February 2020, with case numbers increasing dramatically in March. The search volumes for the terms *coronavirus* and *immune* began to increase slightly in the week of January 26, and they began to increase dramatically in the middle of February. The search volume for the term *vitamin* first began to increase rapidly in the middle of February, but afterward, it repeatedly decreased and increased. The number of cumulative, confirmed cases had a positive correlation with the search terms *coronavirus* (*R*=0.600; *P*<.001), *immune* (*R*=0.763; *P*<.001), and *vitamin* (*R*=0.474; *P*<.001), and the correlations between variables were all mutually statistically significant (Figure 4, Table 4).

**Table 4:** Correlations among search volumes for the terms *coronavirus*, *immune*, and *vitamin*, and the actual number of cumulative, confirmed COVID-19 cases in Italy (over 56 days).

Variable			Cumulative case numbers		Coronavirus		Immune	Vitamin
**Cumulative case numbers**								
	*R*	1		0.931		0.962		0.801
	*P* value	—^a^		<.001		<.001		<.001
**Coronavirus**								
	*R*	0.931		1		0.984		0.842
	*P* value	<.001		—		<.001		<.001
**Immune**								
	*R*	0.962		0.984		1		0.847
	*P* value	<.001		<.001		—		<.001
**Vitamin**								
	*R*	0.801		0.842		0.847		1
	*P* value	<.001		<.001		<.001		—

^a^Not applicable.

**Figure 5:**
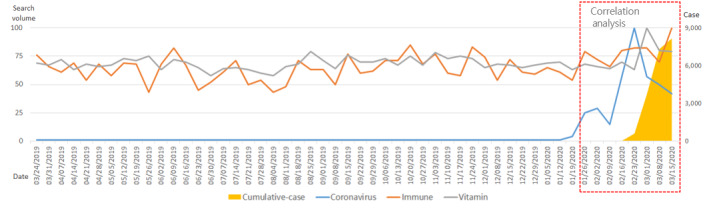
Trends in search volumes for the terms *coronavirus*, *immune*, and *vitamin*, and the number of cumulative, confirmed COVID-19 cases in South Korea over the past year (March 18, 2019, to March 15, 2020).

In South Korea, the number of cumulative, confirmed cases increased rapidly in the middle of February. The search volume for the term *coronavirus* increased earlier—beginning in the week of January 19. After decreasing for a brief period, this search volume again increased rapidly in the week of February 9 and again decreased in March. The search volume for the term *immune* was highest during the first week of March, whereas the search volume for the term *vitamin* was highest during the week of March 15. However, unlike in other countries, the search volumes for these terms did not rapidly increase. The increase in the number of cumulative, confirmed cases had a statistically significant positive correlation with the search volumes for the terms *coronavirus* (*R*=0.359; *P*=.007) and *vitamin* (*R*=0.637; *P*<.001); however, its correlation with the search volume for the term *immune* (*R*=0.254; *P*=.06) was not statistically significant. When analyzing data from Naver, the search volume for the term *coronavirus* increased from the middle of February onward, and the search volumes for the terms *immune* and *vitamin* increased slightly in the beginning of January and increased again in the second half of January and from February 16 onward. All three search terms reached their peak search volumes in the beginning of March, and thereafter, these search volumes began trending downward. When compared with data from Google Trends, the results were similar, and the increasing trend in search volumes for the terms *immune* and *vitamin* that appeared after the occurrence of confirmed cases was slightly clearer (Figure 5, Table 5).

**Table 5:** Correlations among search volumes for the terms *coronavirus*, *immune*, and *vitamin*, and and the actual number of cumulative, confirmed COVID-19 cases in South Korea (over 56 days).

Variable			Cumulative case numbers		Coronavirus		Immune		Vitamin
**Cumulative case numbers**									
	*R*	1		0.359		0.254		0.637	
	*P* value	—^a^		.007		.06		<.001	
**Coronavirus**									
	*R*	0.359		1		0.157		0.242	
	*P* value	.007		—		.25		.07	
**Immune**									
	*R*	0.254		0.157		1		0.368	
	*P* value	.06		.25		—		.005	
**Vitamin**									
	*R*	0.637		0.242		0.368		1	
	*P* value	<.001		.07		.005		—	

^a^Not applicable.

## Discussion

**Figure 6:**
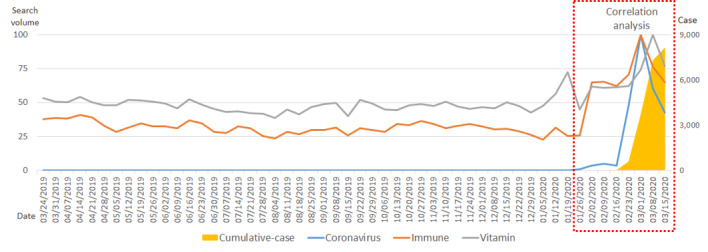
Trends in search volumes for the terms *coronavirus*, *immune*, and *vitamin*, and the number of cumulative, confirmed COVID-19 cases in South Korea over the past year (March 18, 2019, to March 15, 2020) based on data from the Naver portal.

During the COVID-19 pandemic and other pandemics, educational approaches to health and health care are an important problem from the perspective of public health. This study analyzed the increase in the public’s interest in COVID-19 by using social media big data and attempted to gain knowledge concerning what interventions must be implemented from a health and health care perspective. Our findings revealed that increases in search volumes for the terms *coronavirus* and *immune* preceded the actual occurrences of confirmed COVID-19 cases. Moreover, search volumes increased gradually before increasing rapidly at the same time as when a rapid increase in the number of confirmed cases was reported. Search volumes for the term *vitamin* also increased concomitantly. This occurred at the same time as the increase in the number of confirmed COVID-19 cases. Although there were slight differences in the increasing trends among countries, worldwide trends coincided with each other in general. With respect to South Korea, although Google search results did correspond with worldwide trends in a characteristic manner, the data from the domestic portal Naver corresponded more closely with worldwide trends (Figure 6, Table 6).

**Table 6:** Correlations among search volumes for the terms *coronavirus*, *immune*, and *vitamin*, and the actual number of cumulative, confirmed COVID-19 cases in South Korea based on data from the Naver portal (over 56 days).

Variable			Cumulative case numbers	Coronavirus		Immune		Vitamin
**Cumulative case numbers**								
	*R*	1		0.482	0.223		0.629	
	*P* value	—^a^		<.001	.01		<.001	
**Coronavirus**								
	*R*	0.482		1	0.742		0.563	
	*P* value	<.001		—	<.001		<.001	
**Immune**								
	*R*	0.223		0.742	1		0.652	
	*P* value	.01		<.001	—		<.001	
**Vitamin**								
	*R*	0.629		0.563	0.652		1	
	*P* value	<.001		<.001	<.001		—	

^a^Not applicable.

By using search records from its search engine, Google was able to predict the 2009 flu pandemic before the CDC. Similarly, this study also found that the increase in the volume of related search terms preceded an increase in the number of confirmed COVID-19 cases, thereby confirming the validity of using social big data for predicting real-world events. However, there are several possible interpretations for the increased volume of certain search terms. First, this may reflect a rising concern about infectious diseases, such as worries about the outbreak of a disease and demands for knowledge. Second, the results of this study confirmed that in addition to the search volume for the term *coronavirus* increasing, the search volume for the terms *immune* and *vitamin* also similarly increased. This can also be interpreted as an act of disease prevention, which reflects a desire to control situations and prevent disease. During the period of the SARS pandemic, as an act of disease prevention, the number of searches for information on the internet and consultations with experts by people in the United States and Canada who were highly concerned about the disease increased [19]. It is known that increased interest and concern about a disease has a very close relationship with disease prevention measures, and it appears that during a pandemic, increased concern about disease is intimately related to handwashing, personal hygiene and related activities, mask wearing, and the avoidance of public places [19].

In particular, the fact that an increase in search volume preceded the spread of COVID-19 implies that before the complete outbreak of a pandemic, the public has a tendency to be concerned about a disease and engage in corresponding prevention activities. COVID-19 symptoms do not present until 5 to 6 days after SARS-CoV-2 infection, and the incubation period can be as long as 14 days [20]. Taking this into account, the results of this study—those showing that an increase in search volumes directly precedes a rapid increase in cases—reflects the coping mechanisms of a public that has already been exposed to COVID-19 and has been experiencing early warning symptoms. Regardless, before the onset of a full-blown pandemic, supplying prior education and information on health care and health should be the central task of public health departments during these situations.

It cannot be ruled out that an increase in search volumes related to COVID-19 reflects an increase in simple interest. Even if this is the case, it is important to place importance on the dissemination of proper knowledge. The control of a disease’s outbreak cannot rely solely on the efforts of public health specialists. Coping behaviors and prevention measures that are achieved through an increase in knowledge among the public are critically needed [21,22]. In particular, behavioral measures such as social distancing are an effective strategy for coping with an epidemic. However, it is only possible to achieve this through the cooperation of a well-informed public [22]. It is certain that Wuhan residents who moved to other cities without knowing whether they were infected with SARS-CoV-2 contributed to the early spread of COVID-19 [23]. In addition, homeless people and those of similar social classes that have little access to medical information have been identified as potentially dangerous groups that might be transmitters of the disease [24]. Therefore, the propagation of health care information through mass communication methods is emerging as an issue that is more important than ever before. Therefore, in an epidemic or pandemic situation, a variety of strategies are needed to use social media and the internet to alleviate people’s fears and discomfort and increase the public’s awareness of proper health measures.

Alongside the COVID-19 pandemic, not only did the search volumes of the terms *immune* and *vitamin* increase, the number of news reports related to COVID-19 that contained these keywords increased as well (Multimedia Appendix 1). Moreover, in addition to an increase in interest in food products such as elderberry, which are known to be beneficial in enhancing the immune system, because these news reports were related to SARS-CoV-2 (Multimedia Appendix 1), they add to the proof for the public’s increasing interest in immunity following the spread of COVID-19.

The interests of the public are always inextricably tied to the media [25]. As such, rather than supplying sufficient information on the subjects that the public are interested in, news outlets have a tendency to instead provide distracting and shocking content [26]. During a pandemic, web-based media outlets focus on reporting related news, which causes worry about the disease spreading rapidly among the public [27]. Such an anxious and worried public has a tendency to search for health information that cannot be trusted [28]. In particular, it has already been reported that incorrect health information, rather than accurate health information, tends to spread on social networks [29], and this can also cause people to adopt improper health measures [30]. Moreover, despite there being information that is important to understand, during pandemics and similar emergency situations, it is likely that the public will miss certain important information about disease prevention amid the flood of information, and the likelihood of people adopting false beliefs or actions will also rise. The public’s interests and fears concerning COVID-19 can lead to various negative actions such as the panic buying of health products and the aggressive exclusion of certain racial groups, which, in the end, has a negative effect on preventing the spread of SARS-CoV-2 [31,32]. In contrast, during the SARS pandemic, it was known that a certain amount of worry and panic had a positive effect on preventative actions [33], which suggested that because of knowledge deficiencies and areas of irrational risk perception, mediation was needed [33]. Moreover, research on a section of the United States showed that in 2009, newspaper reports on the H1N1 flu had a positive effect on people’s desire to seek prevention measures.

The internet can be used to disseminate information across the globe, and it supplies individually tailored and interactive information. As such, it is an extremely appropriate platform for promoting public health interventions [34,35]. In fact, it is known that information searches conducted through social networks such as Google, YouTube, and Facebook have a positive effect on gaining knowledge about how to prevent infectious diseases, which results in people taking preventative measures [36]. However, as mentioned earlier, the chances are very high that current internet culture will be applied negatively to the COVID-19 pandemic. Within this context, interventions that can assist people in finding information through search word matching and help with actually preventing and coping with the spread of the disease are a key issue with regard to the expansion of proper health literacy. Although such measures can be considered beyond the scope of public health science, during a disaster situation, health authorities must make active interventions to prevent the spread of a disease and do so from a public health science and health information perspective. It is clear that doctors and health care workers must strive to provide accurate information to the public.

It is a known fact that products such as vitamins are helpful for strengthening one’s immune system, and they may have a positive effect on decreasing the rates of infections and deaths caused by SARS-CoV-2 [37,38]. However, although a lack of vitamins can become a medical problem, there is no evidence that an overconsumption of vitamins prevents the transmission of COVID-19. It is known that in seriously ill patients, high doses of vitamin C can be helpful for treatment [39]. However, although it has medical uses, this does not mean that the public should be taking vitamin C to prevent the contraction of COVID-19. The interests of the public, including those of the media, are not necessarily based on accurate medical science. When SARS-CoV-2 emerged, the media disseminated a high volume of reports on immunity and vitamins. Since articles on immunity and vitamins were written mainly about SARS-CoV-2, the public might have believed that vitamin C could prevent SARS-CoV-2 infection. However, although these articles may increase people’s knowledge about self-care related to COVID-19, they can also increase incorrect health literacy. There is also a possibility that such articles will be used for commercial purposes. In the initial stages of the COVID-19 outbreak, the public’s interest in how to protect themselves from infectious diseases, such as by strengthening one’s immune system, grew rapidly. Moreover, an infodemic [40] of misinformation, which spread through various media, became an obstacle to managing public health [41].

During the COVID-19 pandemic, delivering accurate information to the public and correcting false information is the responsibility of experts. Our study confirmed that during the initial phase of the COVID-19 crisis, the public’s desire and actions of strengthening their own immune systems were enhanced. Therefore, when considering our results in conjunction with earlier findings, it is clear that the initial stage of the spread of an infectious disease is the period in which methods for strengthening accurate knowledge about a disease become the most effective. Moreover, these results also verify the importance of intervention strategies that are meant to prevent the spread of an infectious disease during the early stages.

## Appendix

Multimedia Appendix 1 Supplementary tables and figures.

## Abbreviations

CDC: Centers for Disease Control and Prevention
NRF: National Research Foundation of Korea
SARS: severe acute respiratory syndrome
WHO: World Health Organization
