# Supplementary material for: Public Interest in Immunity and the Justification for Intervention in the Early Stages of the COVID-19 Pandemic: Analysis of Google Trends Data
Source: J Med Internet Res. 2021 Jun 18;23(6):e26368. doi: 10.2196/26368 (PMC8216330; doi:10.2196/26368)
Supplement: Multimedia Appendix 1 [file jmir_v23i6e26368_app1.docx]

Table S1

Related queries for immune based on Google web

| Rank | Related queries* |
| --- | --- |
| 1 | Coronavirus immune system |
| 2 | Immune to coronavirus |
| 3 | Coronavirus symptoms |
| 4 | Black people immune to coronavirus |
| 5 | Symptoms of coronavirus |

Table S2

Related queries for vitamin based on Google web

| **Rank** | **Related queries*** |
| --- | --- |
| ***1*** | Vitamin c coronavirus |
| ***2*** | Coronavirus vitamin d |
| ***3*** | Vitamin c corona |
| ***4*** | Vitamin d covid |
| ***5*** | Vitamin covid19 |

Figure S1

Network analysis for immune

Through the news analysis, the relationships between words were visualized with a network. Green collar means place, blue means agency, and yellow means keyword. The numerical value for each word indicates the degree of relevance based on the searched word, and the connection of each line means the association between objects. The analysis is visualized and provided by the Korea Press Foundation, and the data is extracted from news articles of all organizations registered as media in South Korea.

Target period: 1.21. 2020~3.15.2020

Figure S2

Network analysis for vitamin

Through the news analysis, the relationships between words were visualized with a network. Green collar means place, blue means agency, and yellow means keyword. The numerical value for each word indicates the degree of relevance based on the searched word, and the connection of each line means the association between objects. The analysis is visualized and provided by the Korea Press Foundation, and the data is extracted from news articles of all organizations registered as media in South Korea.

Target period: 1.21. 2020~3.15.2020

Figure S3

Cell plot time series visualization between the COVID-19 cumulative confirmed cases, daily new cases, search volume of coronavirus, immune, vitamin, zinc, elderberry, propolis (2020.01.20~2021.01.24).

Each column is an variables of the figures for a selected search term (y axis) updated each week (x axis).
For example, color represents relative density. For example, red indicates a period of relatively more cumulative confirmed cases and daily new cases, and a higher search volume. * Search in each country's language.
